# Relationships within aphids Cinara (Cupressobium) (Hemiptera) based on mitochondrial and nuclear DNA sequences

**DOI:** 10.1007/s13353-013-0184-7

**Published:** 2013-11-28

**Authors:** Roma Durak, Dorota Lachowska-Cierlik, Sławomir Bartoszewski

**Affiliations:** 1 Department of Invertebrate Zoology, University of Rzeszow, Zelwerowicza 4, 35-601 Rzeszów, Poland; 2 Department of Entomology, Institute of Zoology, Jagiellonian University, Gronostajowa 9, 30-387 Kraków, Poland; 3 Department of Biochemistry and Cell Biology, University of Rzeszow, Zelwerowicza 4, 35-601 Rzeszów, Poland

**Keywords:** *Cinara* (*Cupressobium*), Cytochrome oxidase I, Ecology, Elongation factor 1-α, Phylogeny

## Abstract

The relationships between *Cinara* (*Cupressobium*) aphids inhabiting woody parts and leaves of conifers belonging to Cupressaceae have been studied using a mitochondrial gene (COI) and a nuclear gene (EF1-α). Based on the COI sequences, genetic distances between species ranged from 5.6 % between *Cinara (C.) tujafilina* (del Guercio) and *Cinara (C.) juniperi* (De Geer) to 10.5 % between *C. (C.) tujafilina* and *Cinara (C.) mordvilkoi* (Pašek). Genetic distances among EF1-α sequences were lower and showed from 0.1 % between *C. cupressi* and *C. juniperi* to 2.3 % between *C. tujafilina* and *C. mordvilkoi.* Molecular phylogenetic trees were constructed using the Bayesian inference (BI) phylogenetic analysis and maximum parsimony (MP) criterion. Phylogenetic trees obtained based on COI and EF1-α marker genes created two sister clades. Our results indicate that *Cinara* (*Cupressobium*) are a monophyletic group of aphids. Phylogenetic relationships amongst *Cupressobium* aphids do not result from the association with the host plant, but from the feeding site on the host plant or an ability to change the microhabitat on the plant. As closely related species inhabit similar microhabitats on different host plants, it suggests that the host switching is the main mode of speciation in this subgenus.

## Introduction

Aphids belonging to *Cinara* (Hemiptera, Sternorrhyncha, Lachnidae) most often infest woody parts and leaves of Pinaceae and Cupressaceae (Eastop 1972; Blackman, Eastop 1994). The species have simple development cycle (they are monoecious), which enables all generations to develop on one species of host plant. Most of those insect species, classified in *Cinara* subgenus (*Cinara*) are associated with Pinaceae (Blackman, Eastop 1994), a plant family occurring mainly in cool and moderate climates of the northern hemisphere. The host plant species infested by *Cinara* (*Cinara*) consist of the following genera: *Picea* A. Dietr., *Pinus* L., *Larix* Mill, *Pseudotsuga* Carriere, *Abies* Mill., *Cedrus* Belon ex Trew, *Pseudolarix* and *Tsuga* Carriere. The cypress family (Cupressaceae) includes nearly 150 species in 30 genera, occurring mainly in warm climate, e.g., *Juniperus* L.*, Thuja* L.*, Chamaecyparis* L., *Cupressus* L., *Thujopsis* L.f., *Platycladeus* L.*, Microbiota* Kom. genera. These plant genera are infested by aphids from *Cinara* (*Cupressobium*) subgenus (Blackman, Eastop 1994). They can be classified into subgenera according to the following morphological features: the length of dorsal HT I (first segment of the hind tarsus) and the number of subapical hairs on processus terminalis (PT) (Blackman, Eastop 1994).

An interesting phenomenon among *Cinara* infesting the Pinaceae family is the fact that many aphid species can be associated with one plant species. Blackman, Eastop (1994) report that over 100 *Cinara* species are associated with *Pinus sp*. As many as 14 *Cinara* species are trophically associated with *P. edulis* Engelm, while five species are associated with *P. monophylla* Torr., Frem (Voegtlin, Bridges 1988; Favret, Voegtlin 2004). The *Cinara* related to Cupressaceae are a different case, as one aphid species can infest plants belonging to various genera or can be monophagous and associated with one plant species only, while the plant species is also infested by a small number of other *Cinara* species. Blackman, Eastop (1994) report 20 *Cinara* infesting the Cupressaceae family. As all *Cinara* species are very similar, morphological data are not sufficient for adequate phylogenetic analysis (Foottit 1992; Watson et al. 1999).

*Cinara* genus was used by Normark (2000) to determine the evolution course of the Lachnidae family based on the nuclear sequence of elongation factor EF-1α and cytochrome oxydase COII. Also, phylogenetic relationships between the *Cinara* infesting pine trees in southwestern United States based on elongation factor EF-1α and cytochrome oxydase COI (Favret, Voegtlin 2004) were analyzed.

An attempt to define phylogenetic relationships between various *Cinara* species infesting Pinaceae indicated a closer relationship between species occupying similar feeding sites than between those infesting the same host plants (Favret, Voegtlin 2004). On the other hand, Eastop (1986) indicates clear relationships between aphid phylogenesis and their host plants. Some theories highlight the main role of host plants in the process of differentiation and speciation of insects, including aphids (Winkler, Mitter 2008; Peccoud et al. 2010). Due to problems with in-situ monitoring in spite of their large size when compared with other aphids, Cupressaceae-infesting *Cinara* are rarely used in relationship analysis in families or lower units (Ortiz-Rivas, Martinez-Torres 2010). No research on phylogenetic relations between Cupressaceae-feeding species has been conducted so far. Hence this paper aims at exploring relationships between *Cinara (Cupressobium)* species infesting Cupressaceae and defining whether *Cinara (Cupressobium)* are associated closer with a plant species or with their feeding site or microhabitat, determining the type of speciation of these aphids: cospeciation with host plant or by host shifts.

## Materials and methods

### Taxon sampling

Specimens of *Cinara (Cupressobium) cupressi* (Buckton), *Cinara (C.) juniperi* (De Geer), *Cinara (C.) mordvilkoi* (Pašek), *Cinara (C.) fresai* (Blanchard) and *Cinara (C.) tujafilina* (del Guercio) species were collected from 2007 to 2009 from *Juniperus communis*, *J. scopulorum, Thuja occidentalis* and *T. orientalis* (*Platycladus orientalis*) in Poland. They were then preserved in 99.8 % ethanol and stored at −20 °C at the University of Rzeszow, Department of Invertebrate Zoology, Rzeszow, Poland. The COI and EF1-α sequences of *C. cupressi*, *C. juniperi*, *C. mordvilkoi*, and *C. fresai* species obtained during this study were deposited in GenBank (Table 1). We also used COI and EF1-α sequences available from GenBank for *Cinara (Cinara)* and *Rhopalosiphum padi* (Linnaeus) and *Acyrthosiphon pisum* (Harris), belonging to Aphididae as outgroups. All aphid species covered in this study are presented in Table 1, with annotations pertaining to the origin of sequences (this study or GenBank) and GenBank accession number. All voucher specimens are preserved in the Department of Invertebrate Zoology, University of Rzeszow, Poland.

**Table 1:** Aphid species analyzed, classified following Heie and Wegierek (2009), host and GenBank accession numbers of COI, EF1-α gene fragments obtained in this study and those that were already available through GenBank

Tribe	Species	Host plants	Feeding site^a^	GenBank acc. no.	
				COI	EF1-α
Cinarini	*Cinara cupressi*	*Thuja occidentalis*	branch, trunk	JQ247997	JQ248000
	*Cinara fresai*	*Juniperus scopulorum*	branch, trunk	JQ247996	JQ247998
	*Cinara juniperi*	*Juniperus communis*	branch, needle	JN190924^8^	JQ247999
	*Cinara mordvilkoi*	*Juniperus communis*	twig, root	JN190923^8^	JQ248001
	*Cinara tujafilina*	*Thuja orientalis*	twig, root	EU151496^9^	FM174684^4^
	*Cinara anelia*	*Pinus sp.*		EU701607^1^	
	*Cinara atlantica*	*Pinus sp.*		EU701608^1^	
	*Cinara cedri*	*Cedrus sp.*			FM174683^4^
	*Cinara coloradensis*	*Picea sp.*		EU701613^1^	
	*Cinara edulis*	*Pinus edulis*			AY472023^6^
	*Cinara formosana*	*Pinus sp.*		GU978790^2^	
	*Cinara glabra*	*Pinus ponderosa*			AF163870^5^
	*Cinara laricis*	*Larix sp.*		JX034917^3^	
	*Cinara longipennis*	*Abies holophylla*			GU457845^7^
	*Cinara occidentalis*	*Abies sp.*		EU701619^1^	
	*Cinara pergandei*	*Pinus sp.*		EU701621^1^	
	*Cinara picea*	*Picea sp.*		JQ916814^3^	
	*Cinara pinea*	*Pinus sylvestris*		JQ916782^3^	AF163871^5^
	*Cinara ponderosae*	*Pinus ponderosa*			AF163872^5^
	*Cinara shinji*	*Pinus sp.*		GU978787^2^	
	*Cinara strobi*	*Pinus strobus*			AY472033^6^
	*Cinara terminalis*	*Pinus monophylla*			AY472035^6^
	*Cinara wahtolca*	*Pinus edulis*			AY472037^6^
	*Cinara watsoni*	*Pinus sp.*		EU701624^1^	
Aphidini	*Acyrthosiphon pisum*			EU701283^1^	FM174698^4^
Rhopalosiphini	*Rhopalosiphum padi*			EU701893^1^	FM174699^4^

Numbers indicate sequences from previous studies:^1^ Foottit et al. (2008), ^2^ Lee et al. (2011), ^3^ Chen et al.. (2012), ^4^Ortiz-Rivas & Martínez-Torres (2010), ^5^ Normark (2000), ^6^ Favret, Voegthlin (2004), ^7^ Kim et al. (2011), ^8^Durak (2011), ^9^ Durak et al. (2008).

^a^based on Blackman and Eastop and our own observations; twig=0.5-2 cm diameter, branch=2-8 cm diameter.

### DNA extraction, polymerase chain reaction amplification and sequencing

Phylogenies were derived using data from DNA fragments of genes, namely partial COI and partial EF1-α. The DNA was extracted from a single aphid with a standard phenol-chloroform procedure. The DNA was then PCR-amplified with LCO1490/HCO2198 primers (Folmer et al. 1994), which give about 400–650 bp of the COI gene from the mitochondrial genome. The EF1-α of 760–770 bp was amplified with primers EF3 and EF6 (von Dohlen et al. 2002) and a newly designed primer pair EF3b (5’ GTGAAATCGGCAGCACCCT 3’) and EF6b (5’ CACAGAGATTTCATCAAGAACATGAT 3’).

PCR reactions were carried out in 50 μl reactions containing 1 μl DNA (0.4 ng/ml), 1.5 μl of each primer (10pM), 0.5 μl of Taq DNA polymerase (5U/μl), 5 μl of buffer 3 (Expand Long Template PCR System, Roche), 1 μl of 10 mM dNTPs and ultra-pure water. The temperature profile for the amplification of the COI gene fragment included an initial denaturation step at 95 °C for 2 min followed by three cycles at 95 °C for 30 s, 47 °C for 30 s, 72 °C for 1 min 10 s and 32 cycles at 95 °C for 30 s, 51–53 °C for 30 s (depending on primer set), 72 °C for 1 min 10 s and a final extension period at 72 °C for 10 min. Amplification products were resolved by electrophoresis in 2 % agarose gels. PCR products were cleaned with High Pure PCR Product Purification Kit (Roche) and then sequenced at Genomed service (www.genomed.pl).

### Sequence analysis and phylogenetic reconstructions

Sequences were checked and aligned using BioEdit v.7.0.5.2 (Hall 1999) and ClustalX (Thompson et al. 1997). All alignments were visually verified and manually edited. The sequences were also verified for protein-coding frame-shifts using Mega 4.0. in order to avoid pseudogenes (Tamura et al. 2007) and compared with sequences from GenBank with a Blast search. The Akaike Information Criterion in MrModeltest 2.3 (Nylander 2004) in conjunction with PAUP* (Swofford 2002) were used to determine the best-fitting nucleotide substitution model. The GTR+I+G model was chosen for COI and the GTR+G model for EF1-α (Hasegawa et al. 1985). Two methods for phylogeny reconstruction were used – Bayesian inference (BI) and maximum parsimony (MP). BI was run using MrBayes 3.1 (Huelsenbeck, Ronquist 2001; Huelsenbeck et al. 2001) with one cold and three heated Markov chains for 3,000,000 generations and trees were sampled every 100th generation (according to Hall 2007). Each simulation was run twice. Convergence of Bayesian analyses was estimated using Tracer v. 1.5.0 (Rambaut, Drummond 2003–2009) and appropriate number of sampled trees were discarded as ‘burn-in’, and the remainder used to reconstruct a 50 % majority rule consensus tree. MP was computed using PAUP* 4.0b10. For all MP analyses, heuristic search with tree bisection-reconnection (TBR) branch swapping and random addition sequences, MaxTrees = 500 were conducted with 500 random addition replicates. Gaps were treated as fifth character state. Node support was assessed with the bootstrap technique using 5000 pseudoreplicates and TBR branch swapping. Tree reconstruction was performed separately for each marker and afterward on combined sequences. All trees were generated with TreeView 1.6.6 (Page 1996).

## Results

### Sequence analysis

No polymorphism in COI and EF1-α sequences was detected in specimens belonging to particular species, indicating that COI barcode and EF1-α identities were congruent with morphological species concept. Therefore a single sequence was chosen to represent each species in analyses. COI amplification resulted in a 444 bp product for each aphid species. As in other insects, all COI sequences were abundant in AT. EF-1-α primers generated 760–770 bp products which were then sequenced.

The polymorphism rate of mitochondrial genes was approx. 24 % variable sites and about 14 % parsimony-informative sites. Sequences of EF1-α fragments were slightly more variable (about 32 % variable sites and 21 % parsimony-informative sites).

### Phylogenetic analysis

**Fig. 1:**
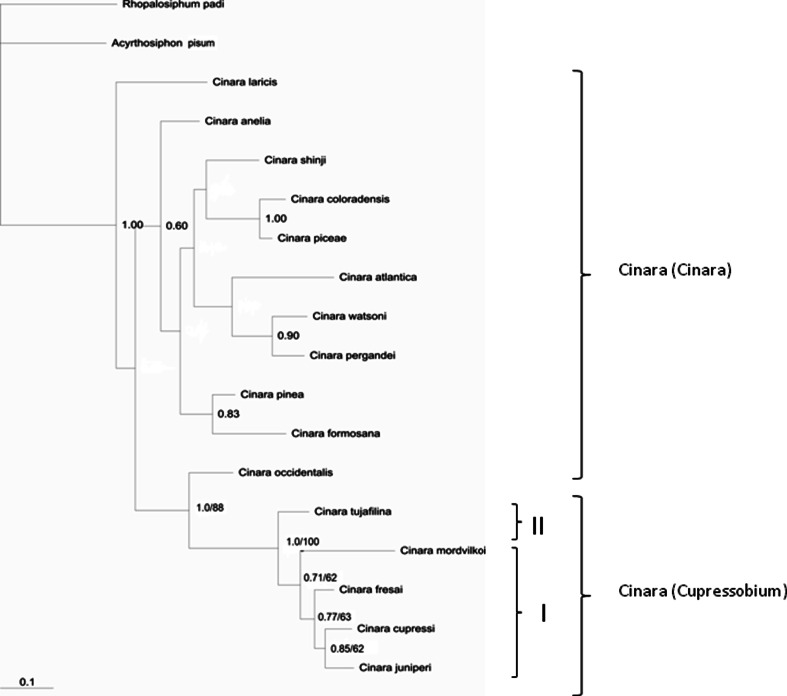
Bayesian tree constructed for COI of *Cinara* (*Cupressobium*). Posterior probabilities of Bayesian inferences/bootstrap values for maximum parsimony are presented on tree branches

**Fig. 2:**
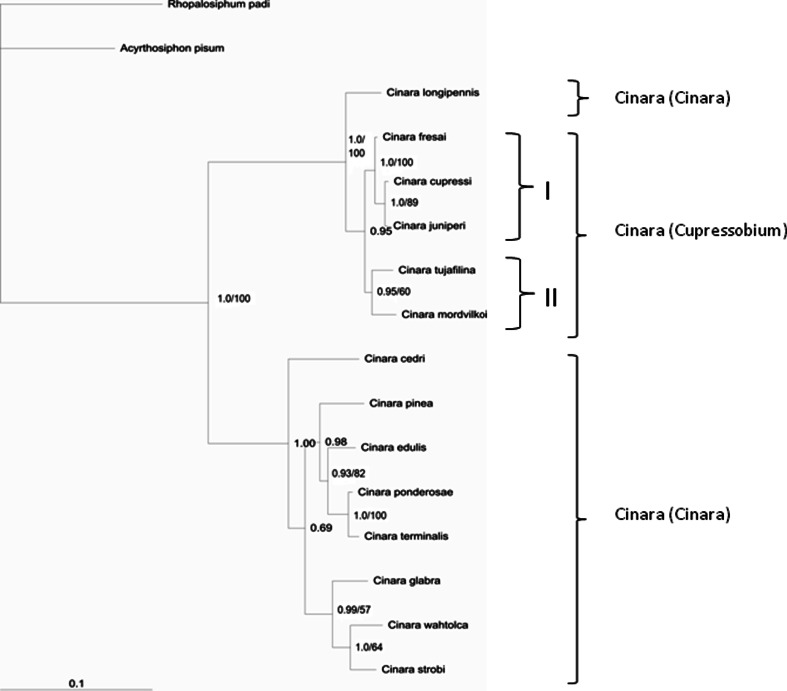
Bayesian tree constructed for EF1-α of *Cinara* (*Cupressobium*). Posterior probabilities of Bayesian inferences/bootstrap values for maximum parsimony are presented on tree branches

MP heuristic searches for COI sequences resulted in one cladeogram (length = 329, consistency index (CI) = 0.5471, retention index (RI) = 0.5300), based on 65 parsimony informative characters. MP heuristic searches for EF1-α sequences resulted in one cladeogram (length = 488, consistency index (CI) = 0.7807, retention index (RI) = 0.8431), based on 172 parsimony informative characters. The GTR+I+G model was chosen for COI (proportion of invariable sites I = 0.5531; gamma distribution shape parameter G = 0.6594) and the GTR+G model for EF1-α (proportion of invariable sites I = 0; gamma distribution shape parameter G = 0.2270), as the best nucleotide substitution model for analyses and pairwise distance calculations. The MP and Bayesian analyses resulted in a congruent topology, so only Bayesian trees are shown in Figs. 1 and 2 (with Bayesian posterior probabilities, PP and MP bootstrap values).

COI barcode genetic distances between species ranged from 5.6 % between *C. tujafilina* and *C. juniperi* to 10.5 % between *C. tujafilina* and *C. mordvilkoi* (Table 2). Mitochondrial distance between species of *Cupressobium* and those of outgroup species was 13.5-17.4 %. The COI proved to be a good barcoding marker as only a 5 % minimum barcoding gap was found within and between species.

**Table 2:** Mitochondrial DNA pairwise distances for all pairs of aphid species studied

	Species	1	2	3	4	5	6	7	8	9	10	11	12	13	14	15	16	17	18
1	*Cinara cupressi*																		
2	*Cinara fresai*	6.1																	
3	*Cinara juniperi*	8.1	6.5																
4	*Cinara mordvilkoi*	10.0	10.0	9.5															
5	*Cinara tujafilina*	6.2	6.5	5.6	10.5														
6	*Cinara anelia*	10.6	10.6	9.7	12.0	10.9													
7	*Cinara atlantica*	13.5	12.4	11.2	14.9	11.8	7.9												
8	*Cinara coloradensis*	10.9	12.5	10.2	14.4	12.0	7.8	9.1											
9	*Cinara formosana*	11.3	11.5	9.9	13.9	11.0	8.3	8.7	9.8										
10	*Cinara laricis*	11.5	10.9	10.0	13.5	11.0	7.0	9.7	9.4	9.3									
11	*Cinara occidentalis*	10.6	8.5	7.2	10.9	9.6	7.4	9.6	9.4	9.2	7.5								
12	*Cinara pergandei*	10.9	10.2	9.4	12.3	10.2	6.7	6.5	7.4	8.0	8.1	7.6							
13	*Cinara picea*	10.5	10.2	9.5	14.9	11.2	6.8	8.6	3.3	8.8	8.8	9.1	6.5						
14	*Cinara pinea*	12.7	12.9	10.3	14.1	13.1	7.1	9.2	8.3	7.1	8.7	9.0	6.6	7.5					
15	*Cinara shinji*	12.3	11.8	11.4	15.5	13.0	8.1	9.2	8.6	8.7	10.0	9.2	6.7	7.1	8.2				
16	*Cinara watsoni*	11.7	12.2	10.4	12.7	11.3	6.9	8.1	7.1	8.8	9.3	8.4	4.1	6.8	7.7	6.8			
17	*Acyrthosiphon pisum*	13.7	14.8	13.8	17.4	14.7	10.9	11.1	11.4	12.6	12.3	11.2	9.6	10.8	11.3	12.3	9.8		
18	*Rhopalosiphum padi*	13.7	13.5	13.8	16.5	14.7	11.4	11.3	14.1	12.0	10.8	11.9	10.7	12.1	13.0	13.3	11.6	10.1	

Genetic distances among EF1-α sequences were lower and amounted to 0.1 % between *C. cupressi* and *C. juniperi* and to 2.3 % between *C. tujafilina* and *C. mordvilkoi.* The genetic distance was 2.1 % between *C. cupressi* and *C. tujafilina*; 2.1 % between *C. cupressi* and *C. mordvilkoi;* 0.3 % between *C. cupressi* and *C. fresai*; 2.0 % between *C. juniperi* and *C. tujafilina*; 2.1 % between *C. juniperi* and *C. mordvilkoi;* 0.2 % between *C. juniperi* and *C. fresai*; 1.7 % between *C. tujafilina* and *C. fresai*. The genetic distance between species belonging to *Cupressobium* and those of outgroup species was 15.6-18.7 % (Table 3).

**Table 3:** Pairwise distances for nuclear DNA sequences from all pairs of aphid species studied

	Species	1	2	3	4	5	6	7	8	9	10	11	12	13	14	15	16
1	*Cinara cupressi*																
2	*Cinara fresai*	0.3															
3	*Cinara juniperi*	0.1	0.2														
4	*Cinara mordvilkoi*	2.1	2.0	2.1													
5	*Cinara tujafilina*	2.1	1.7	2.0	2.3												
6	*Cinara longipennis*	3.2	2.8	3.1	4.0	3.7											
7	*Cinara ponderosae*	12.2	11.8	12.1	12.9	12.8	12.1										
8	*Cinara terminalis*	12.3	11.9	12.2	12.9	12.8	12.2	0.6									
9	*Cinara pinea*	14.7	14.1	14.5	15.3	15.8	14.1	4.2	4.4								
10	*Cinara edulis*	12.2	11.7	12.0	12.7	12.5	12.2	2.5	3.0	4.7							
11	*Cinara wahtolca*	11.9	11.6	11.9	12.8	12.3	12.1	6.0	6.4	8.2	5.9						
12	*Cinara strobi*	12.8	12.5	12.8	13.4	13.0	12.9	5.4	5.1	6.7	5.4	3.0					
13	*Cinara glabra*	11.6	11.2	11.6	12.5	12.0	12.2	5.1	5.5	6.8	5.3	4.1	4.4				
14	*Cinara cedri*	13.0	12.7	13.0	13.8	13.4	12.6	5.9	5.9	7.5	6.7	7.9	7.1	7.2			
15	*Acyrthosiphon pisum*	15.9	16.1	15.7	15.6	16.2	16.5	17.2	17.6	19.8	17.0	16.8	18.0	17.4	16.8		
16	*Rhopalosiphum padi*	18.4	18.3	18.5	18.1	18.7	19.0	18.3	18.8	20.5	18.4	17.8	19.1	18.7	17.9	11.6	

GenBank sequences available for *Cinara (Cinara)* subgenus were also included in phylogenetic tree construction (Table 1). Both phylogenetic trees indicate that *Cinara* subgenus *Cupressobium* form a monophyletic clade sister to *Cinara (Cinara)* (1.0 PP, 88 % bootstrap; 1.0 PP, 100 % bootstrap) (Figs. 1, 2).

The tree based on the mtDNA forms two main clades. Group one (I) includes two well-supported subclades, one containing *C. cupressi, C. juniperi* (0.85 PP, 62 % bootstrap value) and *C. fresai* (0.77 PP, 63 % bootstrap value) and the other one including *C. mordvilkoi* (0.71 PP, 62 % bootstrap value). *C. tujafilina* creates the most external clade (II), sister to group one (I) (1.0 PP, 100 % bootstrap value) (Fig. 1).

The topology of the tree obtained on the basis of EF1-α sequences was similar but showed a closer relationship of *C. tujafilina* with *C. mordvilkoi*. Group one (I) combined *C. cupressi* and *C. juniperi* (1.00 PP, 89 % bootstrap value) and *C. fresai* (1.00 PP, 100 % bootstrap value). Clade two (II) was also well supported and apart from *C. tujafilina* it also included *C. mordvilkoi* (0.95 PP, 60 % bootstrap) (Fig. 2).

All analyses performed indicate that *Cinara* (*Cupressobium*), associated with various Cupressaceae plants, do not form host plant-based clades. However, it was proven that clades encompassed species from the same microhabitat, i.e., the plant part. Both nuclear and mitochondrial data strongly support a clade formed of aphid species infesting above-ground parts of plant, e.g., branches, trunks, and young shoots of various *Juniperus sp.* and *Thuja sp.* (*C. fresai, C. cupressi, C. juniperi*).

The formation of a clade including *C. tujafilina* is very interesting. This clade is very well supported by both nuclear and mitochondrial data analyses. Nuclear data also indicates the strongest similarity between *C. mordvilkoi* and *C. tujafilina*. This is interesting due to the fact that those species do not have common habitat on the host plant, but they change habitats depending on environmental factors. Both species infest lignified plant parts or roots and underground plant parts.

## Discussion

All *Cinara (Cupressobium)* species are closely related, as proven by the genetic distance between them, which fluctuated from 5 to 10 % for COI and from 0.1 to 2.3 % for EF1-α (Table 2, Table 3). Values obtained also enable precise species classification, considering the value of 2 % to be sufficient to separate closely related species with mtDNA (Stern et al. 1997). The obtained range of genetic distances is slightly higher than for *Cinara* (*Cinara*) representatives associated with *Pinus sp*. (Favret, Voegtlin 2004) and similar to that observed between representatives of *Uroleucon* subgenera (Moran et al. 1999).

Relationships between *Cupressobium* species determined based on COI and EF1-α analyses prove that *C. (C.) cupressi*, *C. (C.) juniperi*, and *C. (C.) fresai* are most closely related. They constitute a common clade, strongly supported by mitochondrial and nuclear analyses. Another clade is composed of *C. (C.) tujafilina* and *C. (C.) mordvilkoi* and is well supported by nuclear analyses. Our results for the *Cinara (Cupressobium)* subgenus show that EF1-α is an excellent marker for resolving close relationships between species. Nuclear EF1-α has been used for higher-level studies of insect phylogenetics. Additionally, our results show that EF1-α is also a good marker for resolving relationships at the species and subgenus level. This is supported by previous data indicating EF1-α as a good gene marker for determining phylogenetic relationships between *Megoura* species (Kim, Lee 2008).

The monophyletic character of *Cupressobium* is consistent with the earlier study by Burke et al. (2009), based on studies of symbiotic bacteria such as *Buchnera* and “*Candidatus* Serratia symbiotica” which are associated with Cinarini tribe aphids.

Our studies indicate that *Cinara* (*Cupressobium*) species do not form clades related to their host plants, however, a relationship with their microhabitat on a host plant can be observed.

It seems interesting that the highest genetic similarity is found between species that can infest lignified parts of the host plant, e.g., young shoots, branches, and trunks. Species such as *C. cupressi* and *C. fresai* are associated with many plant genera and their oligophagous features enable them to spread widely around the world, while *C. juniperi* is strictly monophagous and associated with *Juniperus sp*. All species, included in this group (I), infest lignified parts of host plants located above the ground.

Favret and Voegtlin (2004) found that within *Cinara* (*Cinara*) infesting various *Pinus sp.*, a closer genetic similarity is found between species infesting similar microhabitats on plants rather than between species infesting the same species of *Pinus*, but localizing to different microhabitats. *Cinara* (*Cupressobium*) do not form plant-based clades and neither do *Pinus*-related *Cinara* (*Cinara*) (Favret, Voegtlin 2004). This indicates that the evolutionary radiation of this subgenus is not related to plant species and was made possible thanks to the ability to switch host plants. Additionally, phylogenetic relations between the studied aphid species are dissimilar to those obtained from molecular and morphological data on Cupressaceae plants (Gadek et al. 2000; Kusumi et al. 2002). The mechanism of speciation observed for *Cinara* (*Cuppresobium*) involves a host switch rather than aphid-plant coevolution previously shown in *Megoura sp*. (Kim, Lee 2008). The host-switch evolution model is also the most frequently cited speciation model for other aphid species (Moran et al. 1999; Guldemond 1990; Peccoud et al. 2010; Jousselin et al. 2010). A relationship with the feeding site rather than the host plant was also confirmed for *Pterochloroides persicae* (Lassaad et al. 2012).

A separate group (II) of *Cupressobium* are species not related to their microhabitat, which probably results from their ability to frequently change habitats depending on temperature. Such behavior is seen among *C. tujafilina* (which infests *Thuja orientalis*) and *C. mordvilkoi* (which only feeds on *Juniperus communis*) regularly throughout the year (Durak et al. 2008, and unpublished observations). This is also related to a very close symbiotic relationship of those species with ants, which often help them change habitats. However, the ability to infest various plants and easily switch hosts could have been the cause of speciation in phylogenesis of the *Cinara* belonging to both subgenera (Favret, Voegtlin 2004). This indicates their high flexibility facilitating fast spreading (Heie 1994; von Dohlen, Moran 2000; Normark 2000; Heie, Wegierek 2009). This leads to high morphological similarities between species, which often makes them very difficult to identify. Recently molecular analyses have been used to accurately classify those aphid species (Mujtar et al. 2009; Durak 2011; Chen et al. 2012). As with other aphid species, *Cinara* evolved mainly through adaptative evolution involving changes in bionomy, whereas morphological changes are a result of adjustment to infestation of new plant species (Heie, Wegierek 2009).

We suggest that *Cinara* (*Cupressobium*) are a monophyletic group of aphids. Phylogenetic relationships amongst *Cupressobium* do not result from the host plant, but from the site on the host plant or an ability to change the microhabitat. This suggests that the host switching is the main mode of speciation in this subgenus. We prove that host taxonomy cannot be applied to *Cinara* (*Cupressobium*) or *Cinara* (*Cinara*) (Favret, Voegtlin 2004). However, in both cases ecological features of a species are helpful in drawing phylogenetic conclusions. We recommend the EF1-α as an accurate marker gene for reconstructing phylogenetic relationships of lower-level taxa in aphids.
